# Defect Engineering in MoS_2_ Monolayers on Au(111): Insights from Combined Experimental and Theoretical Approaches

**DOI:** 10.1021/acs.jpcc.4c08631

**Published:** 2025-04-23

**Authors:** E. Ascrizzi, M. Nalesso, N. L. Marana, G. Milotti, G. Granozzi, S. Agnoli, A. M. Ferrari

**Affiliations:** † Dipartimento di Chimica, Università di Torino, via Pietro Giuria 5, I-10125 Turin, Italy; ‡ Department of Chemical Sciences, University of Padua, via Francesco Marzolo, 1, 35131 Padua, Italy; § INSTM Istituto Nazionale Scienza e Tecnologia dei Materiali, Padova Research Unit, 50121 Firenze, Italy; ∥ CIRCC Consorzio Interuniversitario per le Reattività Chimiche e la Catalisi, Padova Research Unit, 70126 Bari, Italy

## Abstract

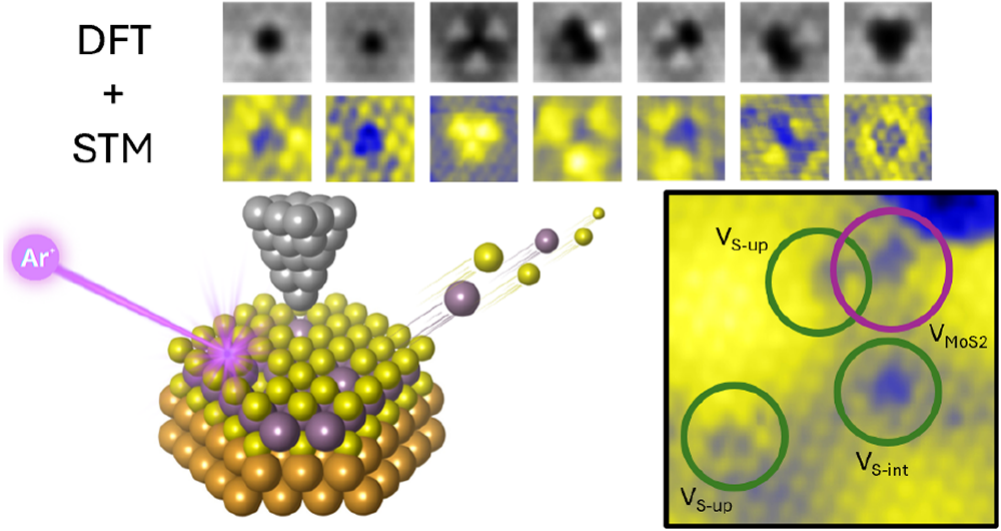

In this study, we present a combined experimental and theoretical study of point defects in MoS_2_ monolayers supported on Au(111). By tuning the experimental conditions, we achieved selective defect formation, paving the way for advanced defect engineering. Density functional theory (DFT) simulations were performed to model both the perfect Moiré superstructure and a variety of defect configurations. This allowed us to precisely identify the experimentally created single- and multiatom vacancies, interpret their contrast in scanning tunneling microscopy (STM), and characterize their electronic properties and effects on the valence band (VB). Our results show that tuning the kinetics of ion bombardment and the chemical environment during annealing treatments can produce different combinations of simple and complex defects. Additionally, we find that the Moiré modulation has minimal impact on the geometric and electronic properties of the surface, suggesting that the defect-engineered MoS_2_/Au surface could serve as a rather general model system to further investigate the electronic and catalytic properties of MoS_2_-based nanomaterials.

## Introduction

In recent years, hydrogen has been identified as a promising candidate as a clean energy vector, and its integration into the energy infrastructure could reduce our dependence on fossil fuels.^1,2^ In this respect, hydrogen should not be produced starting from carbon or hydrocarbons, but from water through electrochemical water splitting.^3^ In this process, molecular hydrogen and oxygen are produced from water electrolysis powered by renewable sources, thanks to the hydrogen evolution reaction (HER) and oxygen evolution reaction (OER), respectively.^4^ The current state-of-the-art catalysts for the electrochemical water splitting reaction are based on Pt/Pd/Ru/Ir metals, but their high costs and scarcity made them inadequate for a rapidly growing mass market.^5^ One possible solution is to improve the catalytic performance of noble metals to balance their high cost, and another is to employ earth-abundant elements. Significant effort has been devoted in the literature to identifying earth-abundant electrocatalysts, with hundreds of compounds proposed, encompassing nearly every transition metal. Interestingly, binary compounds demonstrated to have improved activity with respect to pure metals.^6^ Among possible sustainable catalytic materials, we selected for this study MoS_2_ since it is composed of noncritical raw materials, and its activity in the HER is experimentally and theoretically proven.^7−13^ Moreover, the scientific community has accumulated considerable expertise in the synthesis of high-quality and large-area MoS_2_ films.^14,15^

MoS_2_ thin films are commonly grown on Au(111), as this substrate promotes the formation of highly crystalline and stoichiometric monolayer films rather easily.^16−18^ The Au(111) surface is chemically inert, has a strong electronegativity, and forms a mechanically stable interface with MoS_2_, as demonstrated in previous works.^19−22^ The slight lattice mismatch between MoS_2_ and Au(111) gives rise to a distinctive Moiré superstructure with long-range periodicity. Depending on the growth conditions, this superstructure can adopt either a well-aligned commensurate (11 × 11) superstructure or a small 0.45° rotation may occur between the high-symmetry directions of the Au(111) and MoS_2_(0001) hexagonal lattices.^17,23−25^ The stoichiometric MoS_2_/Au Moiré superstructure has been widely studied. In particular, its formation, stability, and structural properties were explored through both theoretical and experimental approaches.^23−26^

The major inconvenience in using MoS_2_ monolayers as a catalyst lies in the inactivity of the atoms in the basal plane toward water dissociation.^27−29^ The activity of the material is limited to the edges^30^ or to defective sites, such as sulfur vacancies, molybdenum vacancies, or triple vacancies.^11,29,31^

Here, we propose a novel strategy for defect engineering in MoS_2_/Au(111), aiming to precisely control the formation of vacancies and other point defects either through Ar^+^ sputtering or by adjusting synthesis conditions, specifically the chemical potential of sulfur and molybdenum. This strategy is particularly relevant since different types of vacancies are expected to exhibit different physical and chemical properties, as theoretically predicted for unsupported MoS_2_.^10,11,29,31^ By careful tuning of vacancy defect formation, the electronic and chemical properties of MoS_2_ can be tailored for effective material engineering toward improved catalytic performance. Previously, other groups have carried out fundamental studies based on DFT calculations^26^ and scanning probe techniques^32,33^ on defective MoS_2_/Au(111), gaining a detailed insight into the structure and electronic properties of defects. Moreover, ion beam techniques have been clearly recognized as a useful tool for the preparation of defective materials with excellent performance in electrocatalysis.^34−36^ Here, we go further in this direction, reporting a quite comprehensive and systematic catalog of point defects including also nonstoichiometric point defects such as multiatom vacancies consisting of MoS and MoS_3_ units, which were never reported in the literature before.

On the computational side, we performed DFT characterization of the pristine MoS_2_/Au(111) Moiré superstructure and a range of various defective configurations. These calculations provide valuable insights into the structural and electronic properties of this system, while enabling the precise identification of the observed defects. By simulating scanning tunneling microscopy (STM) images, theoretical predictions can be directly compared with experimental data, offering a deeper understanding of defect formation, stability, and their influence on the electronic structure.

## Experimental and Computational Methods

### Sample Preparation and Characterization

The samples were prepared and analyzed in an Ultra-High Vacuum (UHV) system, <10^–9^ mbar. Low-energy electron diffraction (LEED) allowed assessing the proper preparation of the substrate and the long-range order of the overlayer and constant current STM to investigate morphology and structural defects at the nanoscale.

MoS_2_ ultrathin films were grown under UHV conditions (base pressure <1.0 × 10^–9^ mbar) on Au(111) single crystals. The Au(111) single crystal was cleaned by repeated cycles of Ar^+^ ion sputtering (1.5 kV, 1.0 × 10^–5^ mbar of Argon) and annealing to 550 °C. The heating and cooling rate was 1 K/s. MoS_2_ (sub)monolayers were synthesized by filling the chamber with a 2 × 10^–7^ mbar partial pressure of sulfur vapors, produced by sublimating elemental sulfur kept in a glass vial inside a separate chamber connected to the preparation chamber via a gate valve. A hot W filament placed in front of the vial was used to break down the sulfur clusters contained in the vapors. The Au(111) crystal was brought to 450° and exposed to the S atmosphere for five min before depositing Mo from an e-beam evaporator. The Mo deposition rate was determined with a separate experiment by employing the method developed by Cumpson et al.,^37^ with a Mg kα X-ray source, based on the measurement of the signal form the substrate (Au 4*f*) and form the overlayer (Mo 3*d*) as a function of the emission angle. The rate was equal to ∼0.025 ML/min. After 30 min of deposition, Mo evaporation was stopped, and the sample was annealed in a sulfur atmosphere for 15 min at 450° and then at 350 °C in UHV conditions for further 10 min. Scanning tunneling microscopy (STM) measurements were performed with a SPECS SPM Aarhus 150. All STM measurements were conducted in constant current mode at room temperature using an electrochemically etched W tip or a Kolibri sensor. Ultraviolet photoelectron spectroscopy (UPS) measurements were performed with He Iα excitation from a standard helium discharge source, with pass energy (PE) = 2 eV in normal emission (i.e., mainly acquiring the signal related to the Γ point of the Brillouin zone), using a VG Mk II electron analyzer. The Fermi edge of Au(111) allowed us to calibrate the UP spectra binding energy (B.E.).

### Computational Detail and Model Construction

Calculations for the MoS_2_/Au system were carried out, thanks to the Vienna ab initio simulation package (VASP).^38−40^ The recommended projector augmented wave (PAW) potentials were used for all atoms, with a kinetic energy cutoff of 400 eV. PBEsol has been adopted as a functional, given its suitability in describing solid interfaces and its improved capability in computing more accurate lattice constants.^41^ The choice of the functional is crucial, as the overestimation of the lattice constants by many other GGA functionals would modify the overall coincidence of MoS_2_ on Au and the resulting Moiré superstructure. With the PBEsol functional, 4.08 Å was obtained as the bulk gold lattice parameter, as experimentally found.^42^ Dispersion interactions were taken into account using the D3 method^43,44^ because the interaction between MoS_2_ and Au is reported to be based on van der Waals forces.^26^

Bader charges analysis has been performed as implemented in the VASP code,^45^ and STM images have been computed in the framework of the Tersoff–Hamann approximation.^46^ All of the calculations performed on defective systems are spin-polarized.

**Figure 1:**
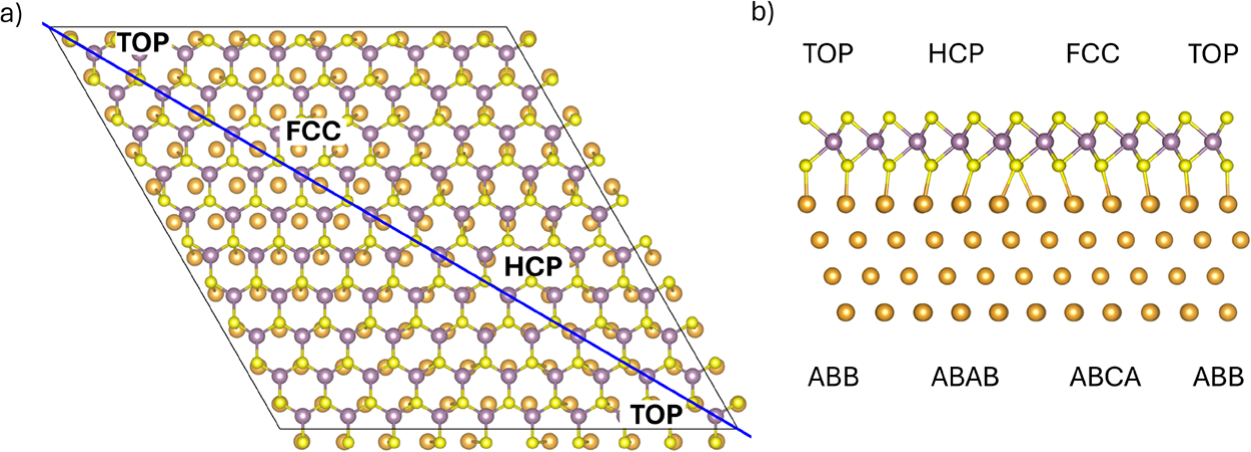
Geometry of the MoS_2_/Au Moiré superstructure, with only the first gold layer visible for sake of clarity in the top view (a), with all four layers of Au considered in calculations in the side view (b). The Mo–S–Au–Au stacking is reported for each high-symmetry domain. Light yellow spheres represent sulfur atoms, purple spheres molybdenum atoms, and dark yellow spheres gold atoms. The black solid line delimits the cell considered for calculations. The plane highlighted in blue was selected for the plots presented in Figure S1.

A layer of MoS_2_ (111) (10 × 10) was placed above four layers of Au (111) (11 × 11), with two lower layers of Au kept fixed at bulk positions during the structural optimization procedure. A regular stacking fashion was modeled for the Mo–S–Au–Au interface, resulting in ABCA, ABAB, and ABB stacking in the fcc, hcp, and top regions (see Figure 1b), respectively, as this stacking is experimentally found to be the most favored.^25^ A vacuum layer of 15 Å has been inserted between the slabs, with the dipole correction along the nonperiodic direction. The resulting structure is shown in Figure 1 and is characterized by a periodicity of 31.7 Å, with no angle between MoS_2_ and the Au substrate lattices, in agreement with experimental results found in the literature.^24−26^ Three high-symmetry domains can be identified in this Moiré superstructure: fcc, hcp, and top. In the fcc domain, both Mo and S atoms are on top of hollow sites of the Au substrate; in the hcp, Mo atoms sit atop of Au atoms of the substrate, while S atoms are on hollow sites; and in the top domain, the reverse is true, with S atoms atop of Au and Mo atoms on hollow sites.

The in-plane lattice parameter of Au(111) is 2.88 Å, whereas the lattice parameter of fully relaxed monolayer MoS_2_(111) is 3.15 Å. The lattice mismatch for the MoS_2_ monolayer in the MoS_2_/Au Moiré model is about 1.05% along both the *a* and *b* directions. Given the large unit cell considered, consisting of 784 atoms, the Brillouin zone was sampled at the Γ point only.

The MoS_2_/Au Moiré superstructure is characterized by an adhesion energy (*E*_ad_) of −90.90 meV/Å^2^, calculated as
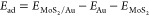
1where *E*_MoS_2_/Au_ is the energy of the MoS_2_/Au Moiré, and *E*_Au_ and *E*_MoS_2_/Au_ are the energies of the optimized Au and MoS_2_ isolated slabs, respectively. The charge transfer from the substrate to the MoS_2_ layer is very small, −18.8 meV per MoS_2_ unit (−2.16 meV/Å^2^), and indicates that the interaction between MoS_2_ and Au is van der Waals, in agreement with previous theoretical studies reported in the literature.^24,26^ The work function of MoS_2_/Au is 4.8 eV, compared to 5.2 eV for the isolated Au slab. This decrease aligns with the charge transfer, but given its limited magnitude, it could also be attributed to a compressive electrostatic effect, as previously suggested.^26^

The formation energies for vacancies (*E*_f_) are calculated as
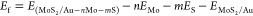
2where *E*_(MoS_2_/Au–*n*Mo–*m*S)_ is the energy of the optimized defective MoS_2_/Au Moiré structure, from which *n* Mo atoms and *m* S atoms are removed according to the vacancy model considered. *E*_Mo_ and *E*_S_ are the energies of S and Mo atoms in the most stable form of the two elements, namely, for S the S_8_ cluster and for Mo the bulk material, and *E*_MoS_2_/Au_ is the energy of the pristine Moiré structure.

## Results and Discussions

**Figure 2:**
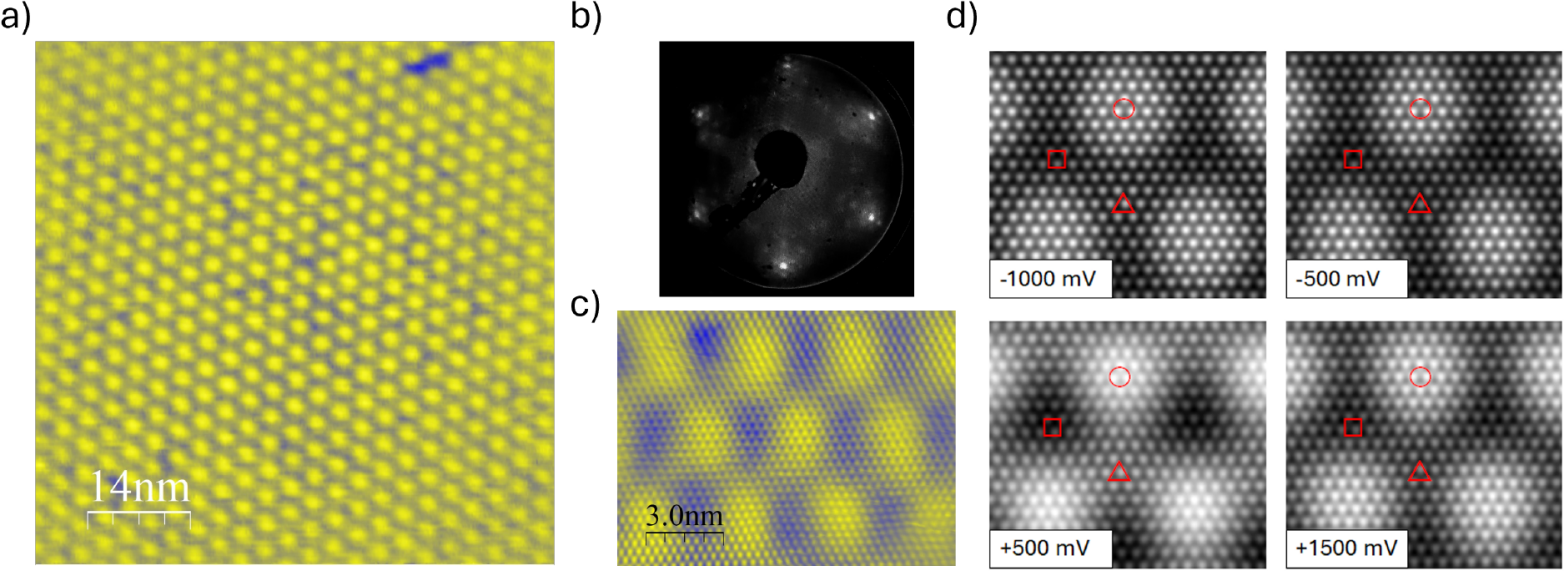
(a) Large-scale topographic STM image of a MoS_2_ island, with its characteristic Moiré pattern (V_SET_ = 1000 mV, *I*_SET_ = 0.2 nA), (b) LEED data (60 eV) showing both the patterns of MoS_2_ and the so-called Au–S “complex phase”, (c) small-area STM topography on a pristine zone. (V_SET_ = 300 mV, *I*_SET_ = 1.0 nA), (d) STM images for the MoS_2_/Au Moiré superstructure computed at different biases. The symbols mark the three high-symmetry domains: □, fcc; △, hcp; and ○ top. Computed images are taken at 2.5 Å above the MoS_2_ layer.

Figure 2a shows a typical constant current STM image of the as-prepared MoS_2_ ultrathin film on Au(111). The electronic density of the MoS_2_ islands shows a characteristic (11 × 11) Moiré pattern. This is caused by a mismatch between the MoS_2_ unit cell and the Au(111) unit cell. The 3.2 nm commensurate supercell is produced by the overlap of (10 × 10) MoS_2_ unit cells on (11 × 11) Au(111) unit cells, as confirmed by LEED measurements (Figure 2b). Given that our synthesis conditions involve an excess of sulfur at high temperatures, the so-called “complex phase” of adsorbed sulfur on Au(111)^47,48^ produces extra diffraction spots that are less intense compared to those related to MoS_2_. A LEED pattern of the S–Au complex phase is reported in Figure S5a, and an STM image portraying its 8.2 Å × 8.8 Å quasi-rectangular unit cell is reported in Figure S5b. Figure 2c shows the small-scale periodicity of the MoS_2_ lattice, made up of bright spots arranged into a hexagonal pattern having a 3.2 Å periodicity, corresponding to the S–S atomic distance, as imaged on the outer layer.

A theoretical analysis of the main structural properties of the three high-symmetry domains (Table S1) reveals that they exhibit very similar characteristics, as expected by the small lattice mismatch and minimal charge transfer between the MoS_2_ film and the Au substrate. The structural parameters analyzed include the lattice parameter *l* (S–S distance), the rumpling of the film Δ*z*_Mo–S_ (computed as *z*_Mo_ – *z*_S_int__, where S_int_ identifies the S atoms in the lower layer, while S_up_ represents the S atoms in the upper layer), interface distance Δ*z*_S–Au_ (height difference between the lower S layer and the topmost Au layer, computed as *z*_S_int__ – *z*_Au_), and Mo–S distance *d*_Mo–S_up/int__, see Table S1. Among these parameters, Δ_S–Au_ is the only feature that slightly differentiates the domains and is the smallest in the top domain. The film rumpling between Mo and S is identical throughout all three high-symmetry domains, indicating that the variations in Δ_S–Au_ are due to the lifting of the upper gold layer, as observed experimentally.^17,23^ This subtle difference induces small localized changes in the charge density and Bader charges, attributed to the interaction between the first Au substrate layer and the lower S layer of the MoS_2_ slab (see Figure S1a and Table S1).

Changes in the surface electrostatic potential above the MoS_2_/Au surface are similarly minor throughout the whole Moiré superstructure (see Figure S1b). Hcp and fcc are characterized by a large zone of surface electrostatic potential that is strongly repulsive for electrons (red), whereas the top domain exhibits a slightly less repulsive potential. This behavior, characterized by minimal structural and electronic variations across the Moiré, is peculiar to this system, as the Moiré modulation significantly influences the electronic and structural properties of other interfaces, such as FeO/Pt or FeO/Au.^49−52^

Simulated STM images obtained at 2.5 Å above the upper S atoms (Figure 2d) closely match the experimental images (compare Figure 2a,c,d). While some differences are observed at varying tunneling biases, the top domain always appears as the brightest. In general, the theoretical description of the MoS_2_/Au Moiré superstructure agrees well with the experimental data from this work and with previous literature studies.^17,23,24,26^

### Vacancies

We simulated 10 different types of vacancies: V_S_up__, V_S_int__, V_Mo_, V_S_2up–up__, V_S_2up–int__, V_MoS_int__, V_MoS_2__, V_Mo_2_S_, and V_MoS_3__, where *up* and *int* identify S atoms extracted from the upper and lower layers, respectively (where not otherwise stated, the S atoms are to be considered *up*). To accurately attribute the experimentally observed defects, we examined a comprehensive range of possible defective structures. Given the large size of the system and the computational cost of the associated calculations, we chose to perform simulations on a single high-symmetry domain of the Moiré, specifically the hcp region. Previous studies by Tumino et al.^26^ on the MoS_2_/Au surface have already investigated certain types of vacancies, showing minimal variation in the formation energy throughout the Moiré. This suggests that defect formation is rather independent from the local registry with the Au substrate. Only defects with no net charge have been considered, i.e., S and Mo are removed from the surface as neutral atoms. *E*_f_ for the considered vacancies are computed as in eq 2, and they are collected in Table 1. Single sulfur vacancies, whether located on the top (V_S_up__) or the bottom (V_S_int__) sulfur layer, exhibit the lowest formation energy; these two types of defects gave comparable *E*_f_. Molybdenum vacancies are significantly more energetically costly than sulfur vacancies (*E*_f_ = 6.30 eV, more than twice the energy required to create a sulfur vacancy, *E*_f_ = 3.22 eV, see Table 1). Furthermore, the formation energy (*E*_f_) increases as the number of atoms involved in the vacancy increases. *E*_f_ data presented in this work are coherent in absolute values and trends with previous works on the MoS_2_/Au and MoS_2_ defective surfaces.^11,26,53^

**Table 1:** *E*_form_ in eV of Vacancies on the hcp Domain of the MoS_2_/Au Moiré Superstructure

defect	V_S_up__	V_S_int__	V_Mo_	V_S_2up–up__	V_S_2up–int__	V_MoS_up__	V_MoS_int__	V_MoS_2__	V_Mo_2_S_	V_MoS_3__
*E*_form_	3.22	3.23	6.30	5.90	6.59	7.71	7.85	9.32	12.12	11.31

**Figure 3:**
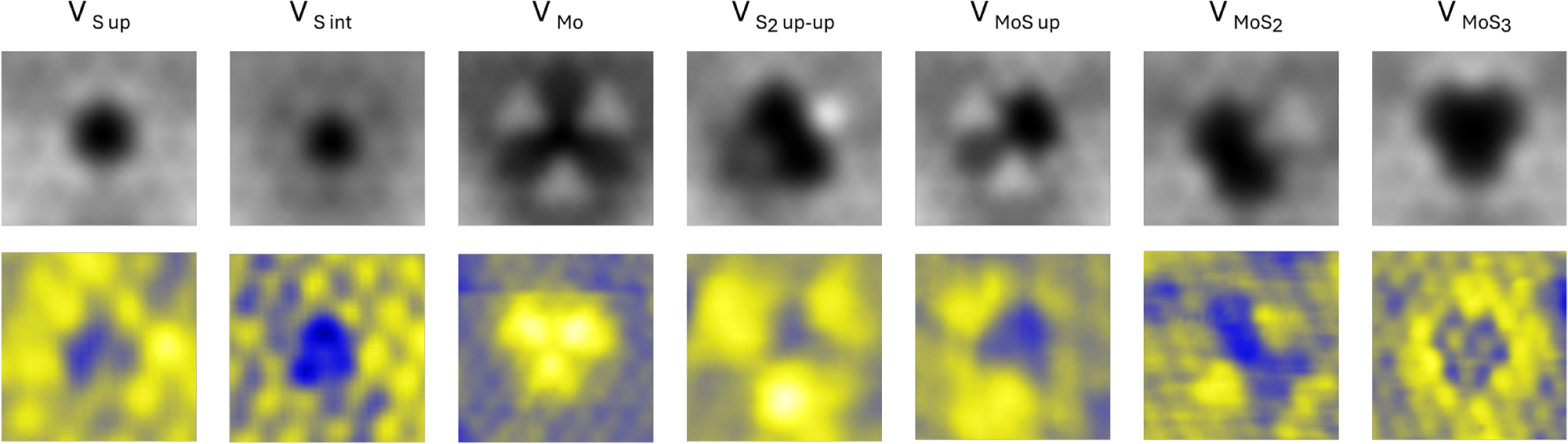
First row: STM images for the MoS_2_/Au Moiré defective structures, taken at bias +1000 mV, 4 Å above the MoS_2_ layer. Second row: experimentally acquired STM images of defects identified on the MoS_2_/Au surface; tunneling parameters are reported in the following. V_S_up__, V_S_int__:V_SET_ = −540 mV, *I*_SET_ = 3.0 nA, V_Mo_:V_SET_ = −1000 mV, *I*_SET_ = 1.5 nA, V_S_2__, V_MoS_2__, and V_MoS_:V_SET_ = 1000 mV, *I*_SET_ = 1.2 nA, V_MoS_3__:*V*_SET_ = −1000 mV, *I*_SET_ = 1.2 nA.

The simulations of the STM topographies of all these defective structures are presented in Figure S2, where the Mo and S atoms have been superimposed to better understand their contributions to the STM contrast. In Figure 3, only the defects that have an experimental counterpart are reported to provide a direct comparison.

Table S2 illustrates the structural and electronic modifications induced by the creation of vacancies, with changes in structural parameters and Bader charges of atoms near the vacancy compared with the pristine structure. In all cases, the formation of vacancies results in a shortening of *l* and *d*_Mo–S_ in the atoms surrounding the vacancy. Among the defects, V_S_ causes the least perturbation of the Moiré structure, which aligns with its lower formation energy (see Table 1). Bader charge analysis reveals adjustments that accommodate the electrons or holes resulting from the removal of S or Mo atoms. Specifically, when holes are left behind, Mo atoms become more positively charged, while S atoms become less negative. Conversely, when electrons are retained within the vacancy, the reverse trend is observed. Interestingly, charge redistribution occurs exclusively within the MoS_2_ layer without any involvement of the Au substrate in the process. In particular, the neutral V_MoS_2__ defect does not alter the overall charge distribution.

### Defect Identification

The defects observed on the pristine and defective samples can be identified by comparing the appearance of the computed defects with high-resolution experimental images. Figure 3 shows a compendium of the calculated and observed defects. Experimentally, defect identification can be carried out following a simple workflow. It starts with counting how many holes interrupt the ideal atomic lattice, which can be achieved by overlaying a semitransparent ideal atomic lattice on top of the experimental image (an example is reported in Figure S6). In this way, the missing atoms can easily be counted, and depending on their number, the contrast of the surrounding atoms allows the identification of the specific vacancy defect. At any tunneling bias, a monatomic sulfur vacancy appears as a single-atom depression (i.e., a dark spot). If the missing atom is on the top layer, the vacancy is accompanied by a homogeneously increased contrast on the neighboring sulfur atoms; if it lies at the interface with the gold substrate, the contrast of the neighboring atoms is not increased. V_Mo_ is easily distinguished as a trilobed structure, characterized by three bright spots that form the vertices of an equilateral triangle with a 5.0 Å long side. We documented a defect consisting of a single-atom depression visible as a dark spot, surrounded by two bright lobes, which entails the loss of the *C*_3_ symmetry axis. We ascribe this kind of defect to a MoS vacancy and, to our knowledge, it had not been previously reported. The lobes possibly result from the redistribution of the electronic density to two pairs of undercoordinated neighboring sulfur atoms, giving enhanced brightness on the two sides. If two atoms are missing, with respect to the pristine basal plane, the vacancy can be either V_MoS_2__ or V_S_2__. V_S_2__ shows two dark depressions on the missing S atoms, whereas the sulfur atom adjacent to the vacant sites and bonded to two coordinatively unsaturated Mo results in very bright. V_MoS_2__ has two vacant sites and increased contrast on one sulfur atom, but the surrounding atoms maintain an apparent corrugation similar to that of the brighter sulfur atom. We also observed a four-atom vacancy, V_MoS_3__, which appears as a sharp, dark trefoil. To our knowledge, this defect was previously only observed by transmission electron microscopy of MoS_2_ film CVD grown on silica.^54^ The strong agreement between the simulated and experimental results, as shown in Figure 3, not only validates the computational setup but also reinforces the interpretation of the experimental observations.

### Strategies for Creating Defects

On a pristine sample, only a few simple defects can be observed. As Table S3 reports, after identification, we systematically counted the defects and normalized their density on the MoS_2_ surface density. Sulfur vacancies are the most common on the as-prepared investigated surfaces. Their density is 2.5 times that of molybdenum vacancies, suggesting that overall the MoS_2_ film is slightly sulfur-deficient. The only other defect that we observed on the pristine surfaces was V_MoS_2__, in a 1:7 ratio with respect to V_S_. Despite the expected lower energy formation of V_S_2__ with respect to V_MoS_2__, it was not observed on the pristine samples. Overall, only 0.12% of the atoms, either Mo or S, are missing from the lattice. The limited density of defects in pristine samples can be intentionally increased with various strategies. We employed sputtering with Ar^+^ ions and annealing in UHV or sulfur/molybdenum excess to modify surface defectivity and composition. After identification, we systematically counted the vacant atoms and normalized their density on the MoS_2_ surface, as summarized in Table S3.

**Figure 4:**
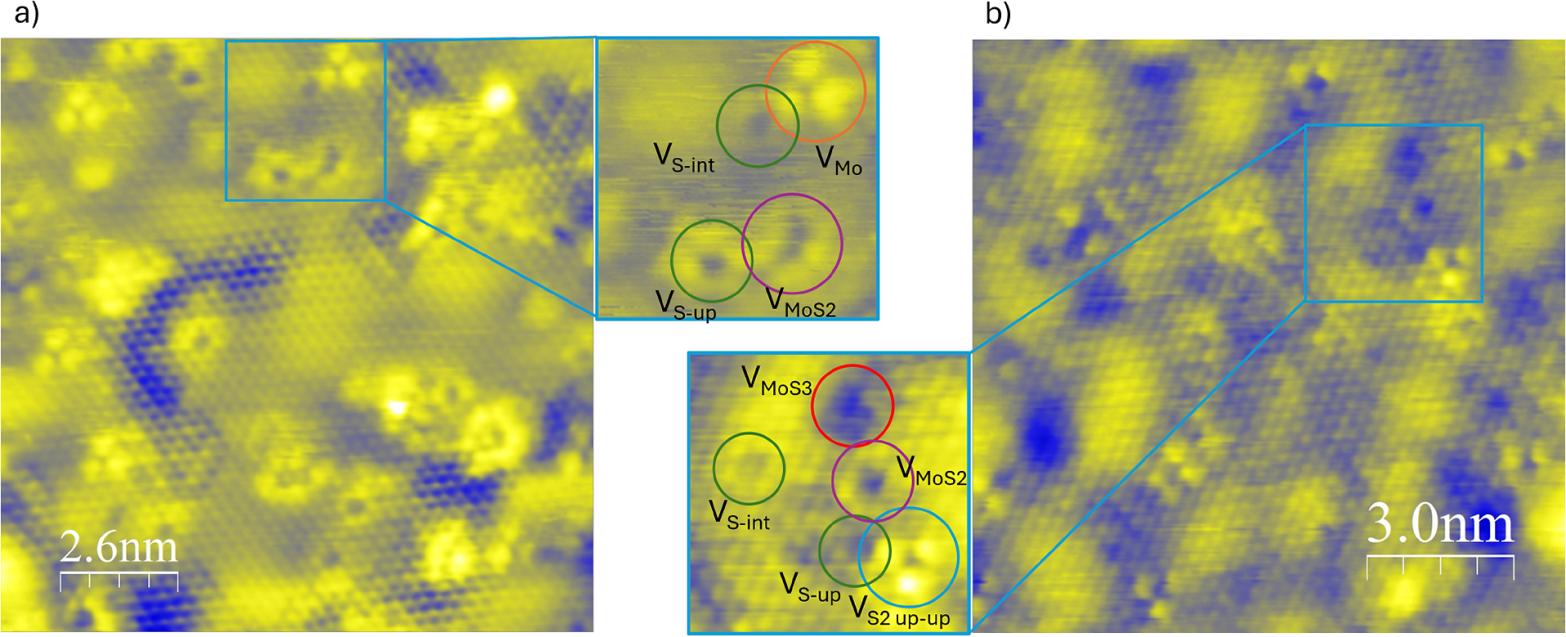
(a,b) STM topographic images of defective MoS_2_/Au(111) surfaces: (a) sputtered sample, showing a damaged but still visible Moiré pattern and various defects (V_SET_ = −800 mV, *I*_SET_ = 2.2 nA); (b) sputtered and annealed sample, with a more regular Moiré pattern and more complex defects (V_SET_ = 1200 mV, *I*_SET_ = 1.2 nA). The insets show in greater detail the observed defects.

Two different dosing times of Ar^+^ ion sputtering were tested, 30 and 10 s, with a 4.7 × 10^11^ ions/s dose. The beam incidence was +45°, and the energy was 0.51 keV. After 30 s of sputtering, the six hexagonal spots around each integer reflection of the Au(111) diffraction pattern disappear, and only the main diffraction feature of the MoS_2_ unit cell remains, indicating the loss of long-range order for the commensurate supercell (Figure S7a). A similar effect is also documented in the STM images that show strong perturbations and in some cases complete suppression of the wavy Moiré pattern. Moreover, occasional clustering and displacement of the material from the MoS_2_ films were observed. Overall, this dose proved to be too disruptive for the film. Careful tuning of the ion fluence on the pristine sample yielded a slightly amorphous MoS_2_ film, an example of which is shown in Figure 4a. This was achieved by reducing the sputtering time to 10 s, preserving the overall Moiré and the LEED pattern integrity (Figure S7b) and still producing many point defects. In these conditions, V_S_ are the most common defects on pristine samples. They are almost triple the V_Mo_, and five times the V_MoS_2__. Ion bombardment caused a sharp increase of the number of defects of all kinds. V_S_ values are more than doubled with respect to the pristine surface, and V_Mo_ increases by a factor of 3. V_S_2__ and V_MoS_3__ are first observed here too; however, single-atom vacancies are preeminent. The ion sputtering procedure increases the amount of missing atoms by three times to 0.3% while preserving the overall morphology and the film. A UHV annealing treatment to 450 °C for 15 min restores part of the lost crystallinity, as the highly ordered LEED pattern shown in Figure S7c shows, and has a strong impact on the density and nature of the defects. An example of this is shown in Figure 4b. It can also provide sufficient energy not only to remove sulfur ions from the lattice but also to promote the diffusion of point defects either to the step edges, as the example in Figure S8 illustrates, or toward each other, where they can recombine. In fact, STM measurements show that the density and complexity of the defects increase after annealing, as 0.8% of the MoS_2_ atoms are vacant, almost seven times the value on the pristine surface. More complex defects could be formed after simple reactions like 2V_S_ + V_Mo_ → V_MoS_2__, or V_S_ + V_Mo_ → V_MoS_. Analogous reactions could justify the formation of V_MoS_3__ defects on a sample characterized by a high defect density.

**Figure 5:**
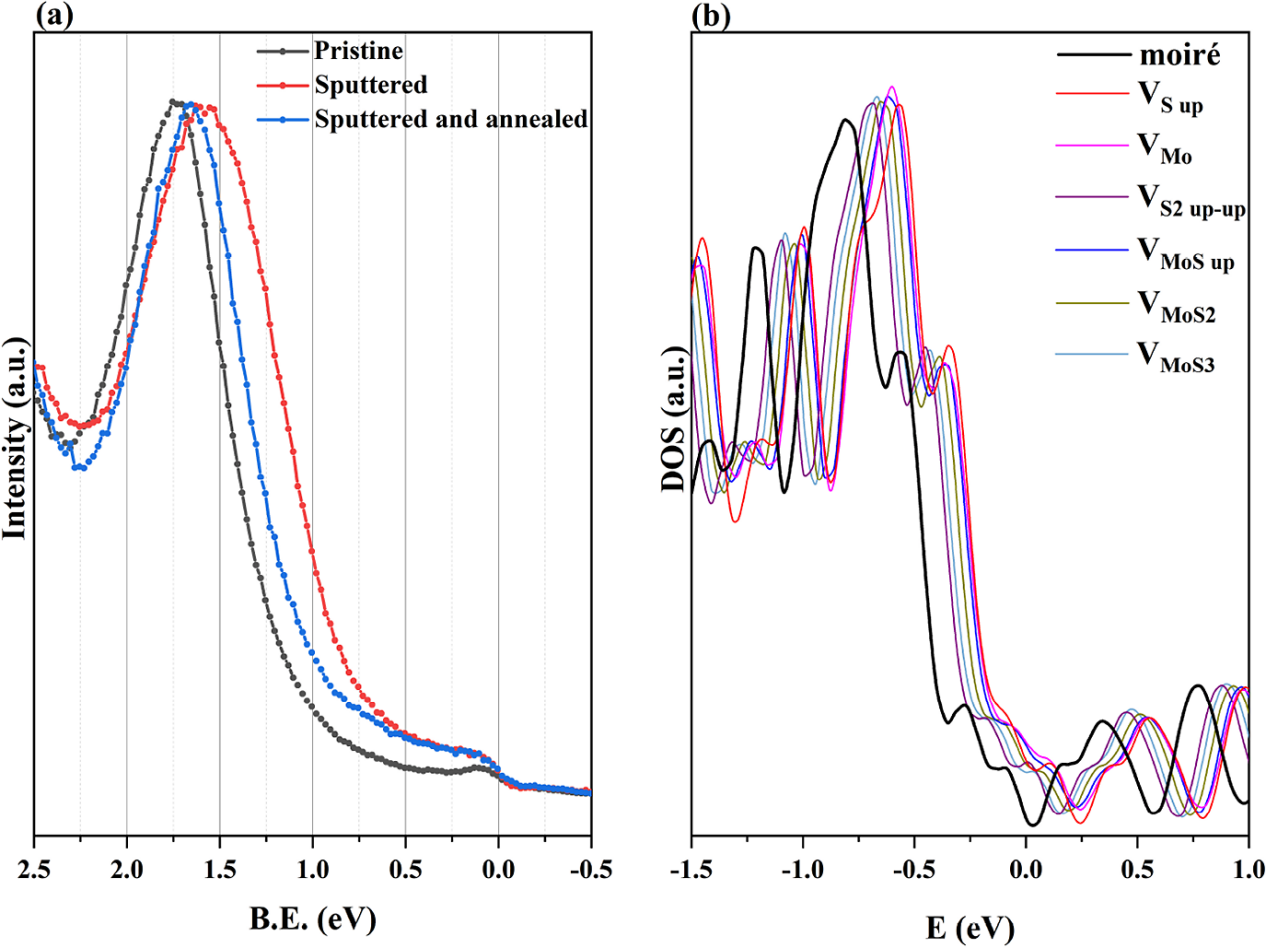
(a) UPS spectra comparing the VB of the pristine film with the sputtered and the reannealed one; (b) computed DOS of the pristine and defective MoS_2_/Au Moiré superstructure. Up and down spin contributions are summed in this plot. The plots have been aligned using the Fermi level (i.e., the work function reversed in sign) of each structure as zero. Only the DOS of vacancies experimentally observed has been reported.

From the computed formation energies (*E*_f_) of the defective structures reported in Table 1, we observe that, with the exception of the V_S_2up–int__ defect, the formation energy of each multiatomic vacancy is lower than the combined formation energies of the corresponding individual atomic vacancies. This suggests that, despite operating outside the thermodynamic control with ion bombardment, once a single-atom vacancy is created, subsequent nucleation of additional defects becomes energetically favorable. For instance, the *E*_f_ of the V_MoS_2__ defect is 9.32 eV, whereas the sum of the formation energies for 2 V_S_ and V_Mo_ is 12.74 eV. Therefore, the defect density of a highly defective sample is a key element for the formation of multiatomic vacancies upon the transfer of energy to the system, for example, by UHV annealing, which further creates more sulfur and molybdenum vacancies. To better understand the effect of the defects on the electronic structure of the film, we performed UPS measurements of the pristine, Ar^+^ sputtered, and annealed samples, and then we calculated the density of states of the pristine sample and of the individual defects. The comparison between the experimental spectra and the computed curves is reported in Figure 5. The experimental data were acquired in normal emission (Γ point of the Brillouin Zone) and show a strong feature at a B.E. of 1.7 eV, in agreement with previous works.^55,56^ After ion sputtering, as a consequence of the formation of defects and partial loss of crystallinity, the valence band (VB) maximum shifts 0.2 eV toward lower B.E.; moreover, the band becomes significantly broader. In fact, the introduction of defects leads to the appearance of states above the VB maximum of the pristine Moiré structure (Figure 5a), contributing to an overall decrease in the n-doping of the MoS_2_ film. After annealing, the valence band spectra change back and again closely resemble those of the pristine sample. However, the centroid of the band remains shifted 80 meV toward the Fermi level, given that even in the annealed samples, the number of defects is higher compared to the initial surface. Analogous results are obtained from the density of states (DOS) calculation of the defective structures found experimentally, as shown in Figure 5b. The calculated DOS correctly reproduces the shape of the experimental UPS spectrum and the energy shift of bands toward the Fermi level, when defects are considered; this is particularly evident for the single vacancies, V_S_up__ and V_Mo_. This behavior is due to S and Mo states that appear in the original gap, as shown in Figures S3 and S4.

When a V_S_up__ is created, two electrons must be accommodated by neighboring atoms; the closest Mo atoms become more perturbed (see charges reported in Table S2) because originally empty 4*d*-Mo levels become occupied, lowering in energy. As a result, donor-like states emerge close to the Fermi level (Figure S4b). Conversely, when a V_Mo_ is created, holes are accommodated on adjacent S atoms, partially withdrawing charge from 3*p*-S levels, which therefore rise in energy and produce acceptor-like levels near the Fermi level (Figure S4c). These features are coherent with vacancy models of freestanding MoS_2_ monolayers.^11,57−59^

Regarding multiatomic vacancies, their effect on the DOS is a combination of what was described for V_Mo_ and V_S_up__. Overall, despite arising from different mechanisms, the effect of defects on the electronic structure of the Moiré is the introduction of new states close to the Fermi level that can be experimentally investigated by UPS. The energy shift between the calculated DOS and the valence band spectra is intrinsic to the calculation method.^60,61^ When comparing theoretical and experimental data, the fixed defect concentration used in calculations has to be considered, set to one defect per unit cell (equivalent to 1.1 × 10^13^ defects per cm^2^).

**Figure 6:**
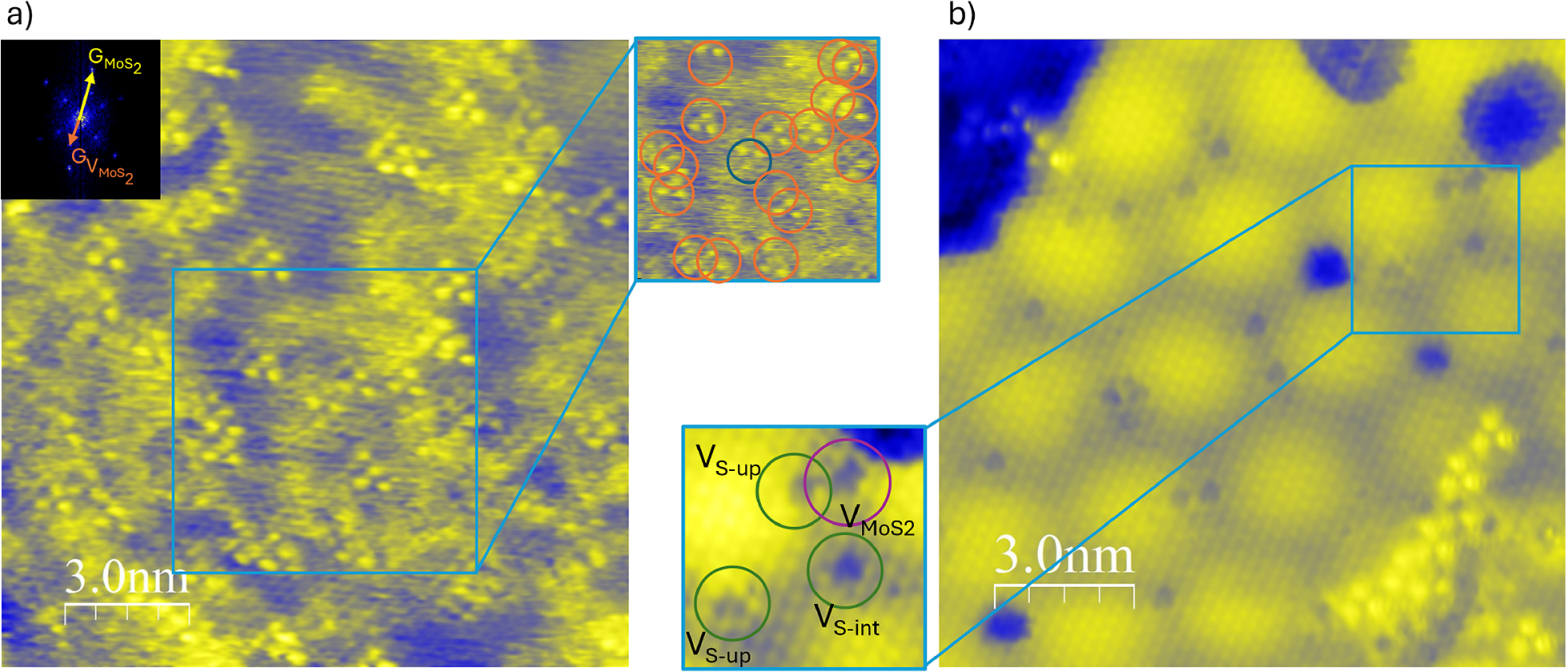
(a,b) STM topographic images of defective MoS_2_/Au(111) surfaces: (a) sample annealed in sulfur-excess conditions, with abundant V_Mo_ (V_SET_ = −500 mV, *I*_SET_ = 2.4 nA). On the right of the image and inset shows a zoom-in of the image. V_Mo_ are circled in orange, and a rotated V_Mo_ is circled in blue. The FFT of the image is shown in the top-left corner, illustrating that both the atomic lattice of the film and V_Mo_ produce reflections in the computed reciprocal space. STM image (b) shows the sample annealed in Mo-excess conditions. Excess Mo has clustered in metallic NPs, and the film has increased defect density (V_SET_ = −540 mV, *I*_SET_ = 3.0 nA).

Ion bombardment proved to be a reliable way to introduce abundant point defects on a MoS_2_ film; however, a similar result can also be achieved by modifying the chemical potential of Mo and S during the synthesis. According to the literature,^26,54^ the formation energy of vacancies is dependent on the sulfur chemical potential (μ_S_) during the synthesis. In sulfur-deficient environments, sulfur vacancies (V_S_, V_S_2__) are the easiest to form. As the environment becomes richer in sulfur, the formation energy of V_Mo_ decreases. For MoS_2_/Au(111), Tosoni et al.^26^ reached the conclusion that above a critical μ_S_, V_Mo_ becomes the stable defect, as its formation energy drops from 4.73 eV in the Mo-rich limit to 2.22 eV in the S-rich limit; on the contrary, for MoS_2_/SiO_2_, Zhou et al.^54^ concluded that V_S_ remains the most stable intrinsic defect even in S-rich environments. We prepared crystalline MoS_2_/Au(111) and then performed a second annealing treatment in a sulfur-rich atmosphere at 380 °C. As Figure 6a shows, without ion bombardment, no multiatomic vacancies were observed, while the density of V_Mo_ sharply increased, reaching 1.6 × 10^13^ defects/cm^2^. The defect density is high enough that the V_Mo_ periodicity can be observed via the fast-Fourier transform filter, and the characteristic Moiré pattern is barely visible. Adjacent vacancies can often be seen sharing one of the three bright lobes, suggesting a configuration for two neighboring V_Mo_. About one defect every 25 is rotated by 60° with respect to the others, appearing as an inverted triangle. The recognizable V_S_ defects are also very sparse, dropping to a concentration similar to that of our pristine samples.

At variance with the previous scenario, a sulfur-deficient synthetic environment is expected to yield a V_S_ abundant MoS_2_ film. In order to test this, we first synthesized pristine MoS_2_/Au(111). Then, 10% more Mo was evaporated on the sample at room temperature and annealed to 450 °C. An example of the film is shown in Figure 6b. The added Mo did not integrate into the MoS_2_ lattice. Instead, it floated as a metal on top of the film over which, upon receiving energy in the form of heat, it diffused and coalesced into nanoparticles. Despite the stability of the Mo–S bond, in fact, the Mo–Mo bond proved strong enough to preferentially aggregate the metal into nanoclusters. Furthermore, the count of defects in this system showed a higher density of V_Mo_ with respect to that of the pristine sample. This suggests that Mo atoms are pulled from MoS_2_ onto the nanoparticles. UHV annealing also generated twice the V_S_ value of the pristine and combined the point defects into more complex ones, for a total of 0.45% missing atoms from the film, about four times the value of the pristine.

## Conclusions

In this work, a novel strategy to obtain vacancy defects with controlled stoichiometry and abundance has been developed.

Ion irradiation and annealing under adjusted conditions (e.g., high sulfur/molybdenum chemical potential) were proven to be effective to obtain materials with a high density and complexity of vacancy defects. These strategies allowed us to increase the percentage of missing lattice atoms from 0.12% in the pristine sample up to 0.8% without compromising film crystallinity. Two previously not observed vacancy defects were first seen by STM imaging, namely, V_MoS_ and V_MoS_3__. Ten types of vacancies were simulated by DFT calculations: V_S_up__, V_S_int__, V_Mo_, V_S_2up–up__, V_S_2up–int__, V_MoS_up__, V_MoS_int__, V_MoS_2__, V_Mo_2_S_, and V_MoS_3__. The comparison between simulated and experimental STM images enabled the precise identification of the observed defects by correlating the electronic contrast and the structural patterns. Both DFT calculations and ultraviolet photoemission spectroscopy experiments show that the defective films exhibit peculiar electronic properties since most types of vacancies create new intragap states, which overall narrow the band gap and induce p-doping. The information provided is crucial for establishing a comprehensive framework for the rational design and optimization of novel MoS_2_-based functional materials, where advanced defect engineering can effectively tailor electronic structures and active sites, thereby enhancing performance, efficiency, and selectivity in electro- and photocatalytic technologies.

## Supporting Information Available

Characterization of the simulated MoS_2_/Au pristine; defective moiré superstructures; computed STM images of all the defective structures simulated; DOS structures for all the systems considered, projected on Mo, S and Au atoms; LEED pattern and STM image of the Au–S complex phase; STM images of the defective sample; experimental defect density for the different considered systems (PDF)
