# Supplementary material for: Defect Engineering in MoS2 Monolayers on Au(111): Insights from Combined Experimental and Theoretical Approaches
Source: J Phys Chem C Nanomater Interfaces. 2025 Apr 23;129(17):8086–95. doi: 10.1021/acs.jpcc.4c08631 (PMC12051453; doi:10.1021/acs.jpcc.4c08631)
Supplement: Supplementary file 1 — jp4c08631_si_001.pdf [file jp4c08631_si_001.pdf]

Supporting Information: Defect Engineering in MoS_2 Monolayers on Au(111): Insights from Combined Experimental and Theoretical Approaches

E. Ascrizzi,[†,‡] M. Nalesso,[†,¶] N. L. Marana,[‡] G. Milotti,[¶] G. Granozzi,[§] S. Agnoli,[¶,‖,⊥] and A. M. Ferrari[*,‡]

†*Contributed equally to this work*

‡*Dipartimento di Chimica, Università di Torino, via Pietro Giuria 5, I-10125 Turin, Italy.*

¶*Department of Chemical Sciences, University of Padua, via Francesco Marzolo, 1, Padua 35131, Italy.*

§*Department of Chemical Sciences, University of Padua,via Francesco Marzolo, 1, Padua 35131, Italy.*

‖*INSTM Istituto Nazionale Scienza e Tecnologia dei Materiali, Padova Research Unit, Firenze 50121, Italy.*

⊥*CIRCC Consorzio Interuniversitario per le Reattività Chimiche e la Catalisi, Padova Research Unit, Bari 70126, Italy.*

E-mail: anna.ferrari@unito.it

Table S1: Properties of the three different high-symmetry domains of the MoS_2/Au moiré superstructure. The lattice parameter l (S-S distance), the film rumpling Δz_{Mo-S} (computed as $z_{Mo} - z_{S_{int}}$, where S_{int} identifies the S atoms in the lower layer, while where S_{up} identifies the S atoms in the upper layer), interface distance Δz_{S-Au} (height difference between the lower S layer and the first layer Au, computed as $z_{S_{int}} - z_{Au}$), Mo-S distance $d_{Mo-S_{up/int}}$, Bader charges $q_{Mo,S,Au}$ are to be intended as mediated for the atoms at the center of each high-symmetry domain.

domain	l[Å]	Δz_{Mo-S}[Å]	Δz_{S-Au}[Å]	$d_{Mo-S_{up/int}}$[Å]	q_{Mo}[e]	q_S[e]	q_{Au}[e]
fcc	3.16	1.56	2.51	2.39/2.40	+1.16	-0.59	+0.004
hcp	3.18	1.54	2.54	2.39/2.40	+1.18	-0.60	+0.004
top	3.12	1.54	2.48	2.38/2.38	+1.14	-0.58	+0.011

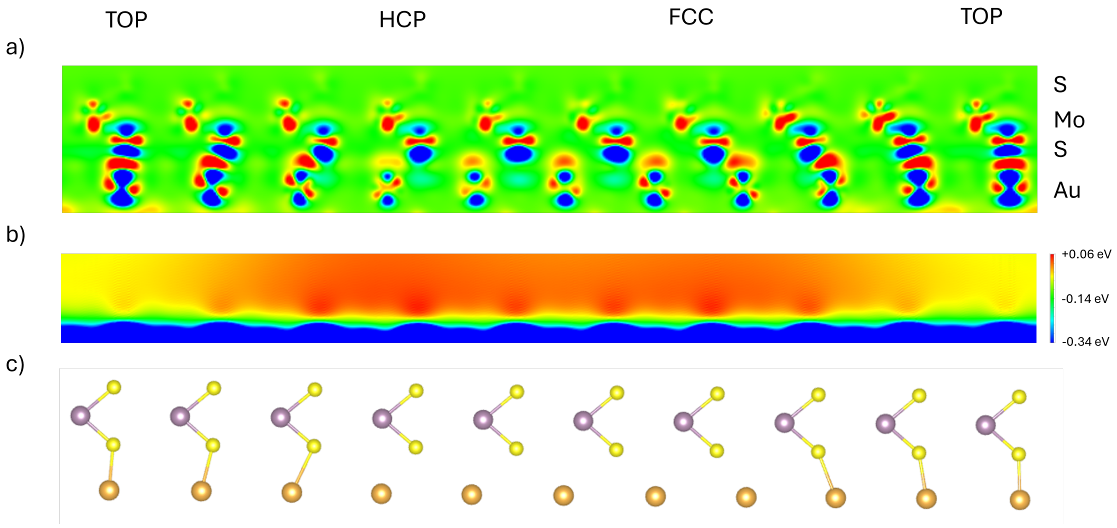

Figure S1: a) Difference electron density map between the MoS_2/Au moiré, the MoS_2 and the Au isolated slabs, for a plane passing through Mo and S atoms of the three high-symmetry domains (the one highlighted in blue in Figure 1 of the manuscript). Regions of electron excess and deficiency are depicted in red and blue, respectively. Only the MoS_2 film and the first layer of the Au substrate are shown in this Figure for sake of clarity. b) Electrostatic potential of the MoS_2/Au surface plotted on a the same plane. c) Atoms cut by the plane that passes through the three high-symmetry domains. Light yellow spheres represent sulfur atoms, purple spheres molybdenum atoms, dark yellow spheres gold atoms.

Table S2: Structural properties of the hcp domain in the pristine and defective MoS_2/Au moiré superstructure. The lattice parameter l (S-S distance), the film rumpling Δz_{Mo-S}, interface distance Δz_{S-Au}, Mo-S distance $d_{Mo-S_{up/int}}$ are to be intended as mediated for the atoms at the center of hcp high-symmetry domain in the pristine case, while in the defective cases we mediated on first neighbour atoms close to the vacancy.

	l[Å]	$\Delta_{z_{Mo-S}}$[Å]	$\Delta_{z_{S-Au}}$[Å]	$d_{Mo-S_{up/int}}$[Å]	q_{Mo}[e]	q_S[e]	q_{Au}[e]
hcp (pristine)	3.18	1.54	2.54	2.39/2.40	+1.18	-0.60	+0.004
$V_{S_{up}}$	3.18	1.50	2.52	2.39/2.36	+1.03	-0.69	+0.004
$V_{S_{int}}$	3.17	1.55	2.52	2.36/2.38	+1.03	-0.67	+0.002
V_{Mo}	3.14	1.57	2.42	2.39/2.35	+1.23	-0.53	+0.008
$V_{S_{2_{up-up}}}$	3.10	1.50	2.51	2.33/2.36	+0.99	-0.69	+0.011
$V_{S_{2_{up-int}}}$	3.09	1.49	2.52	2.39/2.38	+0.83	-0.62	+0.006
$V_{MoS_{up}}$	3.10	1.08	2.89	2.39/2.28	+1.11	-0.48	+0.004
$V_{MoS_{int}}$	3.11	1.49	2.41	2.27/2.35	+1.19	-0.54	+0.007
V_{MoS_2}	3.13	1.14	2.41	2.35/2.27	+1.18	-0.60	+0.007
V_{Mo_2S}	3.16	1.57	2.37	2.37/2.43	+1.24	-0.51	+0.010
V_{MoS_3}	3.14	1.45	2.45	2.39/2.34	+1.09	-0.60	+0.008

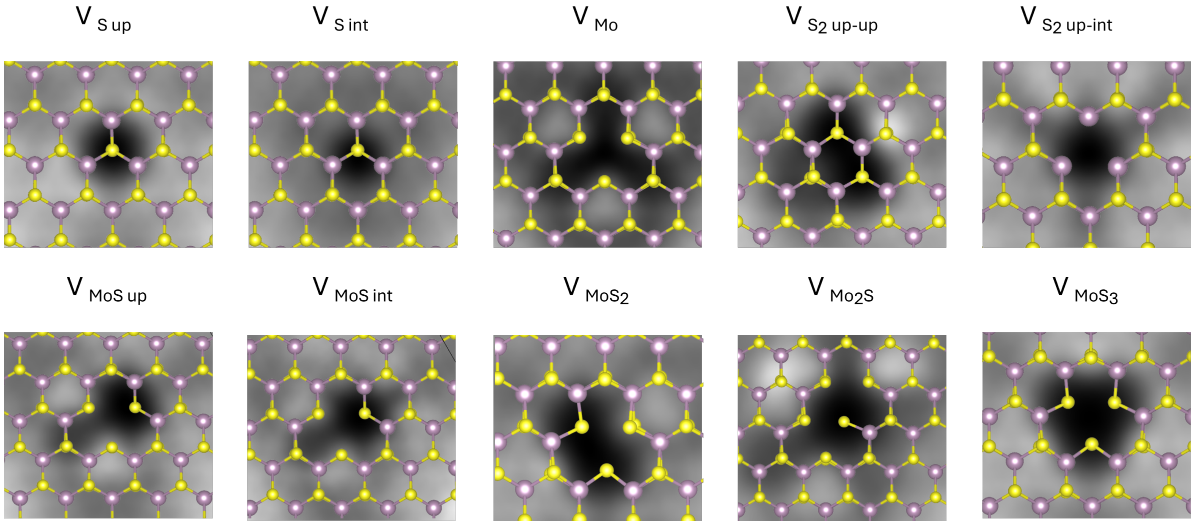

Figure S2: STM images for the MoS_2/Au Moiré defective structures, taken at bias + 1000 mV, 4 Å above the MoS_2 layer. Reticular S and Mo atoms have been superimposed to better attribute contrast features. Light yellow spheres represent sulfur atoms, purple spheres molybdenum atoms.

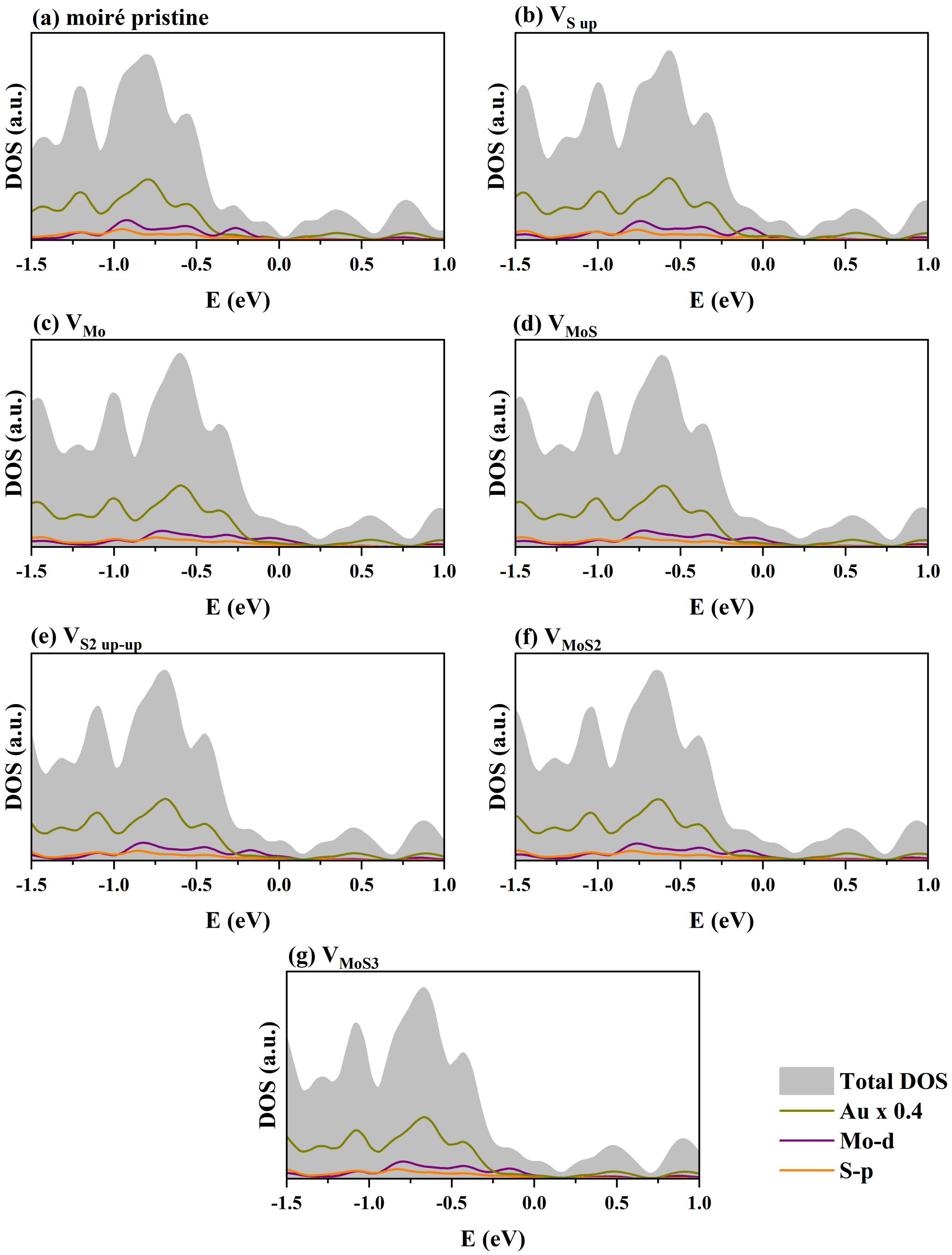

Figure S3: Computed DOS projected on the d, p and d atomic orbitals of Mo, S and Au atoms of the pristine and defective MoS_2/Au moiré superstructure. Up and down spin contributions have been summed in this plot. The plots have been aligned using as zero the Fermi level (i.e. the work function reversed in sign) of each structure. Only DOS of vacancies experimentally observed have been reported.

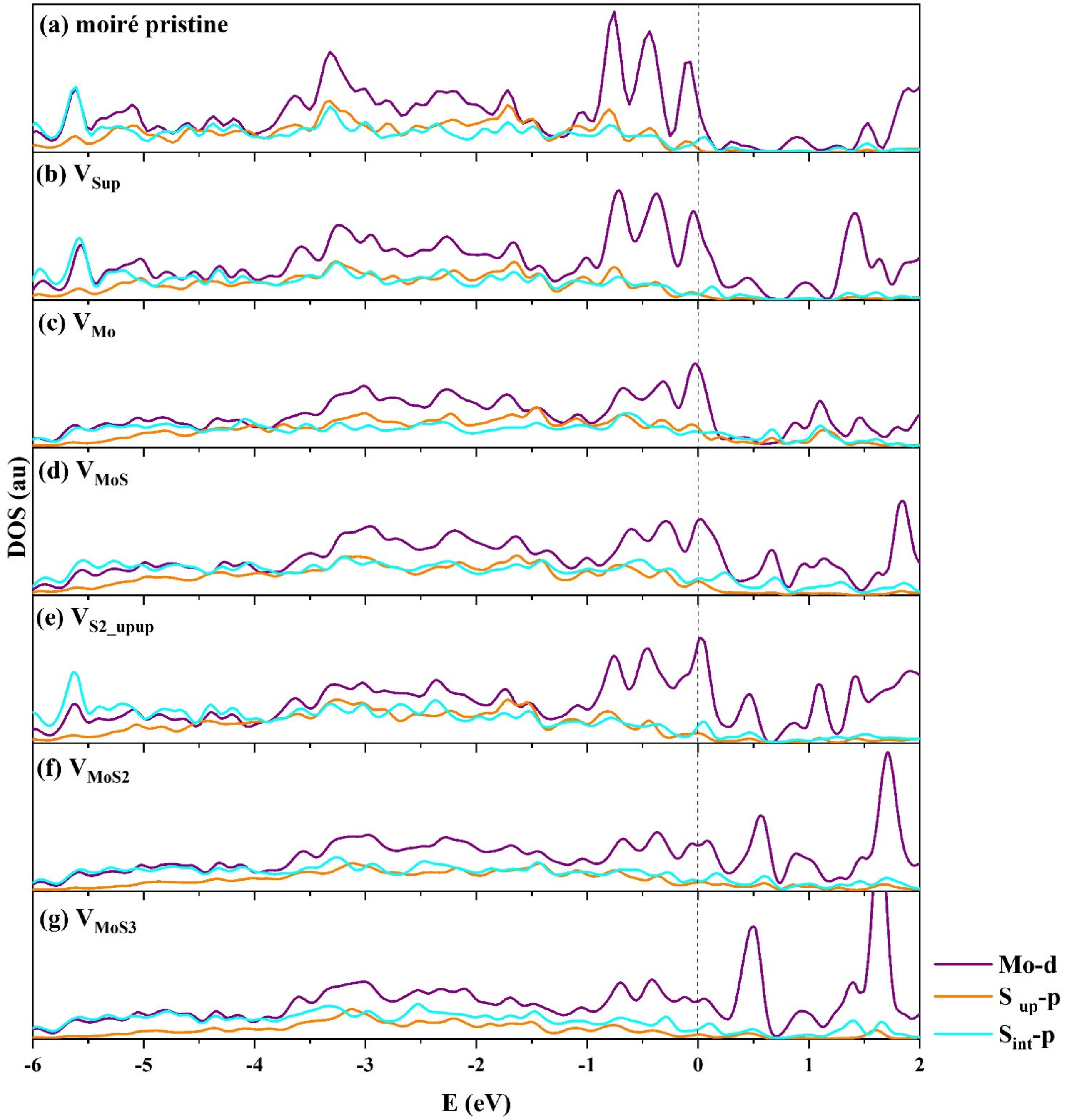

Figure S4: Computed DOS projected on the d and p atomic orbitals of Mo and S atoms of the pristine and defective hcp high-symmetry domains of the MoS_2/Au moiré superstructure. Up and down spin contributions have been summed in this plot. The plots have been aligned using as zero the Fermi level (i.e. the work function reversed in sign) of each structure. Only DOS of vacancies experimentally observed have been reported.

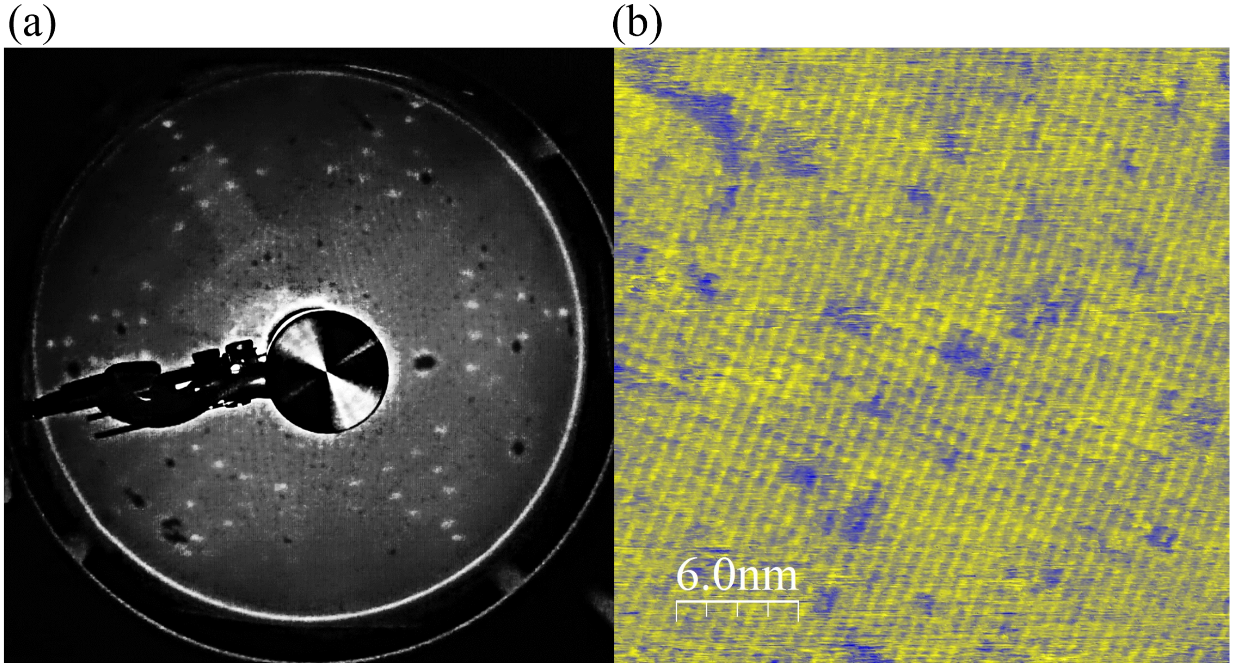

Figure S5: (a)LEED pattern and (b) STM image (V_{SET} = 500 mV, I_{SET} = 1.0 nA) of the so-called Au-S complex phase. It is obtained by exposing the clean Au(111) surface to sulfur vapor doses greater than 3x10^4 L to temperatures above 450 K.

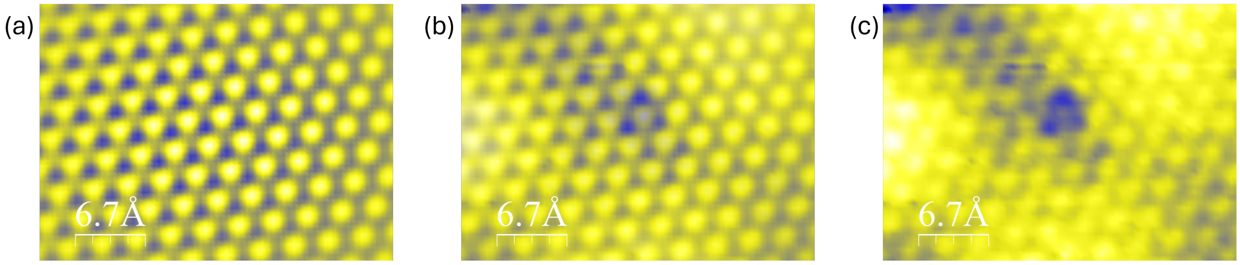

Figure S6: Example of how the number of missing atoms can be counted. The STM image (V_{SET} = -540 mV, I_{SET} = 3.0 nA) of a single $V_{S_{int}}$ amid the MoS_2 lattice was covered by a model of the defect free atomic lattice (a). By increasing transparency of the overlaid model image from to 60 (b) and to 100 (c) the number of missing atoms is easily counted, being one in this case.

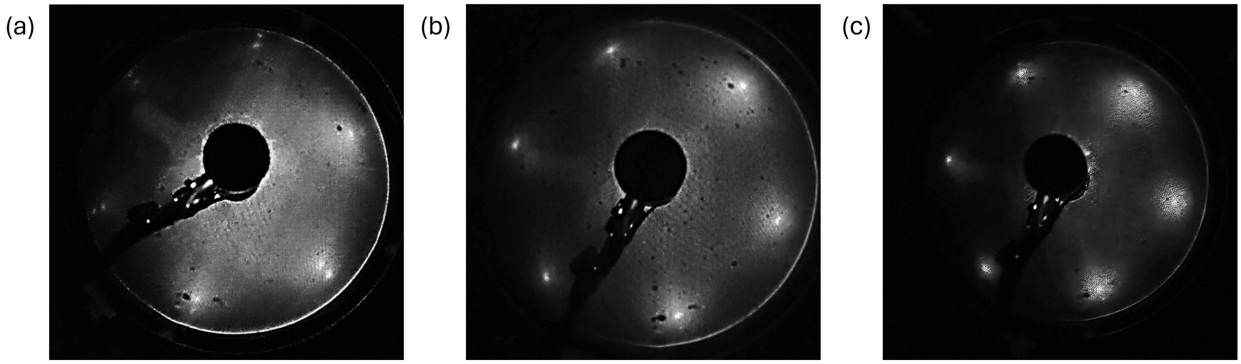

Figure S7: LEED patterns acquired with 60 eV electron kinetic energy in various stages of the sample modification process to achieve higher defect density. In all of them, the S-Au complex phase diffraction spots are gone due to the sputtering. Panel (a) shows that after the 30 s sputtering on a pristine sample the Moiré first order diffraction spots around the main MoS_2 one are gone, indicating total loss of long range periodicity; panel (b) shows that, after the 10 s sputtering on a pristine sample, the Moiré satellite reflections are still visible, with a lower resolution and greater noise than on the pristine, indicating partial loss of long range periodicity; after the 15 minutes UHV annealing to 450°C (c), the 10 s-sputtered sample recovered the satellite spots and the long-range order.

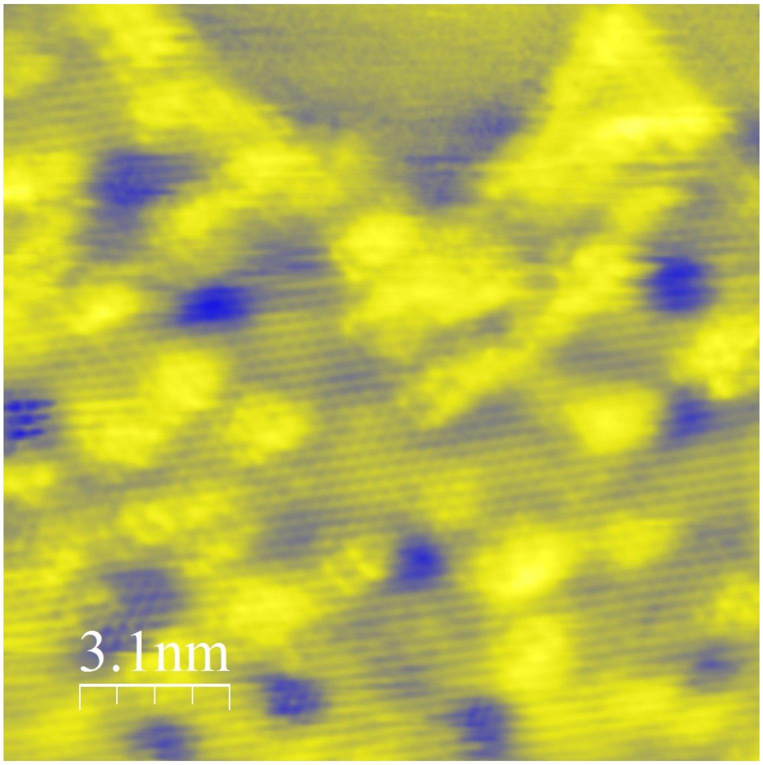

Figure S8: Topography STM image (V_{SET} = -1000 mV, I_{SET} = 1.2 nA) of the sample after Ar^+ ion irradiation and UHV annealing at 450°C. The top area of the image represents the step edge of the MoS_2 film, where defects clustered.

Table S3: Defects density for the different considered systems. All values are in [(numerosity of defects)/cm^2].

	Pristine	Sputtered	Sputtered and annealed	Sulfur Excess
V_S	$(2.3\pm0.2)\times10^{12}$	$(5.3\pm0.2)\times10^{12}$	$(6.6\pm0.8)\times10^{12}$	$(2.3\pm0.3)\times10^{12}$
V_{Mo}	$(9.1\pm1.3)\times10^{11}$	$(2.7\pm0.2)\times10^{12}$	$(2.9\pm0.3)\times10^{12}$	$(1.6\pm1.1)\times10^{13}$
V_{MoS}	-	-	$(3.1\pm0.8)\times10^{12}$	-
V_{S_2}	-	$(4.2\pm0.7)\times10^{11}$	$(6.9\pm1.3)\times10^{11}$	-
V_{MoS_2}	$(3\pm0.9)\times10^{11}$	$(4.2\pm0.7)\times10^{11}$	$(2.5\pm0.3)\times10^{12}$	-
V_{MoS_3}	-	$(6.2\pm1.5)\times10^{10}$	$(9.9\pm1.3)\times10^{11}$	-
Sum	$(3.5\pm0.3)\times10^{12}$	$(9.0\pm0.3)\times10^{12}$	$(1.7\pm0.1)\times10^{13}$	$(1.8\pm0.1)\times10^{13}$
